# The Book World of Medicine and Science

**Published:** 1895-01-12

**Authors:** 


### The Book World of Medicine and Science.

Diseases of the Nose and Throat. By F. De Havilland Hall, M.D. (London: H. K. Lewis.)

The volume is the latest addition to "Lewis' Practical Series," but whether it supplies a genuine want, in view of the numerous text-books on the same subject which have preceded it, is not for us to determine. Judged, however, on its own merits, it appeals to us as a trustworthy and practical treatise, with just enough information in it to supply the reader with the facts he may require, whether he be student or practitioner. Its pages do not weary one with abstruse theoretical questions, and, further, it is guiltless of the mistake of containing too many personal hobbies of treatment, an error which frequently mars the work of specialists. Amongst a number of good sections, that on adenoid growth is perhaps the most successful, and we are glad to see that the author has risen to the importance of the occasion, and has sufficiently impressed his readers with the necessity of recognising the symptoms and taking prompt measures when they are found. The article on diphtheria, which is a subject of at least as much importance, although occupying a good proportion of the pages of the book, is perhaps not treated with the same degree of skill which is observable in other subjects. The reader is confused by the number of alternative methods of treatment, and should his fancy alight on any one in particular, we rather think that without considerable previous practical experience, the directions found scattered through the text would prove lamentably insufficient. Far more valuable to the reader would be the full account of the treatment which Dr. Havilland Hall himself advocates, for, as everyone is aware, it is on the minutiæ that the success or failure of any particular line of treatment depends. The omission of the operative directions for tracheotomy is a bold one in a work which poses as a complete treatise on diseases of the throat, and hardly finds sufficient excuse in the professional apology that the author is a physician and not a surgeon, and that surgical text-books may be referred to if occasion should arise. The book, however, has so much to recommend it that it is perhaps scarcely fair to cavil at what are merely errors of omission.

Dissections Illustrated. Part III. The head, neck, and thorax, with twenty coloured plates and eight diagrams. By C. Gordon Brodie, F.R.C.S. (London: Whittaker and Co. Price 10s.)

This, the last part but one of Dr. Brodie's series of dissections of the human body, well maintains the high standard of excellence initiated in the earlier folios. The plates, which have been drawn and lithographed by Mr. Percy Highley, are peculiarly successful in indicating the depth at which the various structures are situated; a feat of no easy performance, especially in the case of deeply-seated structures in the triangles of the neck. The only objection that one can urge against these illustrated dissections is, that the student's work is made too easy for him, and he may be induced to rely too little on the scalpel and forceps.

Lunacy Law for Medical Men. By Charles Mercier, M.B. (London: J. and A. Churchill. Price 5s.)

The aim of this book is to give to the medical practitioner clear and definite information on the points of Lunacy Law which may come before him. We think it is a pity that the author did not include among the medical men for whom he was writing those who are in charge of institutions for the care and treatment of the insane. To medical superintendents of asylums a knowledge of the Lunacy Law is most essential, and assistant medical officers generally provide themselves with some work on this subject. It would be disappointing to them after obtaining "Lunacy Law for Medical Men" to find that the title is misleading.

Everyone knows that it is to the medical attendant that the relatives of the mentally afflicted naturally fly for guidance in the delicate matter of placing the patient under control. The directions given by Dr. Mercier will be found full and easily followed. An account of the procedure necessary for obtaining the requisite order is given, and warnings are added concerning the examination of the patient and the wording of the medical certificates. Those whose lot it is to peruse many of these documents will be able to appreciate the wholesome advice offered, and will wish that it were more generally acted upon. It is not very flattering to the profession to find that the author deems it necessary to give lessons in orthography, although we must reluctantly admit that there are cases in which a lack of this quality is painfully apparent. We believe that much worry and trouble would have been spared to medical men if they had been impressed with the importance of one caution upon which great stress is laid, viz., never to authorise or assist in the obtaining the signature of an insane person to a document. There are occasions on which it would seem almost cruel to be thus careful, but nevertheless the warning is a salutary one.

The author remarks upon the invidious situation in which the Commissioners place the person receiving a private patient, inasmuch as he is practically made to review the right of a judicial authority to make a reception order upon the certificates laid before him.

Detailed information concerning the legal obligations of the medical practitioner who has charge of an insane person is given. The chapter of which we would speak in special terms of commendation is that in which advice is given to the medical man in the event of his being called to examine a prisoner, and to give evidence concerning his mental condition. Attention to the hints here given will tend to increase the comfort of the witness when in the box and under cross-examination.

The City Diary (Messrs. W. H. and L. Collingridge, 148, Aldersgate Street, E.C.)

This diary has now reached its thirty-second year of publication. It is conveniently arranged for busy people who wish to record facts in as brief a manner as possible, and to have a quite comprehensive reference book at hand. A great deal of postal and City information is to be found in the first 24 pages of this useful diary.

